# Texas

**Published:** 1880-12

**Authors:** 


### TEXAS.

Mr. Montgomery Gibbs, of London, England, the well-known "Anglo-Saxon" of the London Press, one of the proprietors of the Journal of Health, and a frequent contributor to our pages, is now on his way to Texas. He is the editor of the "Englishman's Guide Book to the United States and Canada," the only guide book to America published in Europe. While in America Mr. Gibbs will entirely re-write that work, especially as to Texas and southwestern America, for the benefit of British tourists and settlers. The eminent standing of Longmans & Co., the oldest and most extensive publishers in London, gives to this work, as to all works published by them, the character of authority.

Mr. Gibbs is the author of a series of letters in relation to Texas which, over the signature of "Anglo-Saxon" have recently appeared in the London *Daily News*, the London *Live Stock Journal* and other leading journals, and which have attracted much attention. His visit to the South will include Arkansas and Texas, every part of which States he will personally inspect with a view to the selection of the best fields for British emigration. On his return to England he will deliver lectures in twenty towns of Great Britain on the emigration fields of America. *En route* to Texas he spent some time in Canada, for the purpose of personally examining its claims to the attention of British settlers. So great is the depression in agriculture in the British Islands that thousands of once well-to-do farmers must seek new homes, and Mr. Gibbs has been pressing the claims of Texas upon the people as the most promising field, by reason of the fertility of its soil and the salubrity of its climate.

In the interest of the Journal of Health our associate will contribute to its pages from time to time articles on the climate and health of Texas and southwestern America. It is claimed that the climate of Southwestern Texas is the best in America for arresting, if not for the cure of, diseases of the lungs and throat. Several of its mineral springs are said to furnish a cure for diseases of the kidneys. If these things are true, Texas must become a vast *sanitarium*. The exhaustive letters of our associate upon the subject will be read with interest by thousands of readers.

The results of the visit referred to will be published in the spring of 1881, by Messrs. Longmans, of London, under the title "The Whole Truth About Texas."

